# Direct comparison of gluco-oligosaccharide oxidase variants and glucose oxidase: substrate range and H_2_O_2_ stability

**DOI:** 10.1038/srep37356

**Published:** 2016-11-21

**Authors:** Thu V. Vuong, Maryam Foumani, Benjamin MacCormick, Rachel Kwan, Emma R. Master

**Affiliations:** 1 Department of Chemical Engineering and Applied Chemistry, University of Toronto, 200 College Street, Toronto, Ontario, M5S 3E5, Canada

## Abstract

Glucose oxidase (GO) activity is generally restricted to glucose and is susceptible to inactivation by H_2_O_2_. By comparison, the Y300A variant of gluco-oligosaccharide oxidase (GOOX) from *Sarocladium strictum* showed broader substrate range and higher H_2_O_2_ stability. Specifically, Y300A exhibited up to 40 times higher activity on all tested sugars except glucose, compared to GO. Moreover, fusion of the Y300A variant to a family 22 carbohydrate binding module from *Clostridium thermocellum* (*Ct*CBM22A) nearly doubled its catalytic efficiency on glucose, while retaining significant activity on oligosaccharides. In the presence of 200 mM of H_2_O_2_, the recombinant *Ct*CBM22A_Y300A retained 80% of activity on glucose and 100% of activity on cellobiose, the preferred substrate for this enzyme. By contrast, a commercial glucose oxidase reported to contain ≤0.1 units catalase/ mg protein, retained 60% activity on glucose under the same conditions. GOOX variants appear to undergo a different mechanism of inactivation, as a loss of histidine instead of methionine was observed after H_2_O_2_ incubation. The addition of *Ct*CBM22A also promoted functional binding of the fusion enzyme to xylan, facilitating its simultaneous purification and immobilization using edible oat spelt xylan, which might benefit the usage of this enzyme preparation in food and baking applications.

Glucose oxidase (GO) belongs to the auxiliary activity family AA3_2 (www.cazy.org) and is a flavoenzyme with a tightly but non-covalently bound FAD cofactor^1^. The enzyme oxidizes the anomeric carbon of β-D-glucose using molecular oxygen as an electron acceptor, producing H_2_O_2_ and D-glucono-δ-lactone, which in the presence of water spontaneously hydrolyzes to gluconic acid^2^.

GO is one of the most widely used industrial enzymes^2^, and many commercial applications employ GO from *Aspergillus niger* (NCBI accession: AJ294936) since the properties of this enzyme have been extensively studied^3^. Applications of GO include use in biosensors, biofuel cells, as well as in food products^4^,^5^. Considering applications in food, GO has been used to reduce browning caused by the Maillard reaction^6^; the oxygen scavenging activity of GO and simultaneous production of H_2_O_2_ can also reduce food spoilage^7^,^8^. Moreover, the production of H_2_O_2_ can improve dough stability by forming cross linkages within gluten^9^; production of H_2_O_2_ can also oxidize water-soluble pentosans^10^, which then gelate and increase water uptake and softness of baked products^11^. The introduction of aldonic acids into dough is also expected to reduce stickiness and improve machinability^11^.

Despite the broad range of applications for GO, the effectiveness of GO is restricted by the narrow substrate range of this enzyme and susceptibility to H_2_O_2_ inactivation. Kleppe^12^ showed that less than 10 mM H_2_O_2_ that reduced 20% of enzyme activity oxidized methionine residues within the GO active site, where as 100–200 mM H_2_O_2_ reduced enzyme activity by up to 80%^12^. Similarly, H_2_O_2_ was reported to oxidize surface exposed methionine residues and a phenylalanine in the active site of a pyranose oxidase (Family AA3_4) from *Trametes multicolor*^13^. Commercial preparations of GO can include catalase to overcome GO inactivation by H_2_O_2_. However, this solution is not practical for applications that benefit from H_2_O_2_ production by GO.

Where broader substrate range is beneficial, alternatives to GO may be found among the oligosaccharide oxidases from family AA7 (www.cazy.org), such as the carbohydrate oxidase from *Microdochium nivale* (MnCO)^14^, and gluco-oligosaccharide oxidase from *Sarocladium strictum* (GOOX)^15^,^16^,^17^. Like GO, both MnCO and GOOX oxidize the anomeric carbon of carbohydrate substrates through an FAD reduction mechanism; however, in these cases, the FAD cofactor is bi-covalently linked through cysteine and histidine residues. Furthermore, these enzymes display a comparatively broad substrate profile, ranging from xylose, and galactose, to malto-, cello- and xylo-oligosaccharides^14^,^16^,^18^. Similar to MnCO, GOOX oxidizes oligosaccharides more effectively than monosaccharides^16^. To date, thirteen GOOX variants have been created through site-directed mutagenesis^16^,^19^, where one variant (Y300A) shows higher activity on monosaccharides (including glucose and xylose) compared to the wild-type GOOX. The Y300A variant also retains activity on oligosaccharides and displays reduced substrate inhibition^19^.

MnCO activity has already been reported to increase dough elasticity and consistency^20^ and was tested as a substitute for GO in baking applications^21^. Nevertheless, MnCO was inactivated by H_2_O_2_ during substrate turnover^22^, although the extent of H_2_O_2_ inactivation was not reported.

The comparatively broad substrate range of Y300A along with reduced substrate inhibition make it a suitable alternative to GO particularly in applications that comprise mixed carbohydrates. Accordingly, this study directly compared the substrate profile and H_2_O_2_ inactivation of the *A. niger* GO and the Y300A variant of GOOX. The Y300A variant was also fused to *Ct*CBM22A, a carbohydrate-binding module from *Clostridium thermocellum*, generating *Ct*CBM22A_Y300A to facilitate enzyme purification through binding to edible oat fibre.

## Materials and Methods

### Materials

Glucose, xylose, oat spelt xylan (OSX), and beech wood xylan were purchased from Sigma (St. Louis, USA) while cellobiose and maltose were purchased from BioShop Inc. (Ontario, Canada). Cello-oligosaccharides and xylo-oligosaccharides (XOS) were purchased from Megazyme (Wicklow, Ireland), and mixed XOS (DP-2–7, 95% pure) were obtained from Cascade Analytical Reagents and Biochemicals (Oregon, USA). Wheat bran hemicellulose and propoxylated wheat bran hemicellulose were kindly provided by Prof. Yaman Boluk (University of Alberta, Canada). Gluconic acid was obtained from Thermo Fisher Scientific (Massachusetts, USA). *Aspergillus niger* glucose oxidase (Cat. no. G0543, with ≤0.1 units/mg protein catalase) and bovine liver catalase were purchased from Sigma (St. Louis, USA).

To prepare insoluble OSX, 2 g OSX was suspended in 200 mL of 50 mM Tris-HCl pH 8.0 for 48 h at room temperature and then washed three times. The washed OSX was then filtered through a 0.45-μm nylon membrane, and the dry weight of the retentate was measured.

### CBM fusion synthesis

The *Ct*CBM22A sequence with its native connecting loops (10 amino acids at the N-terminus and 7 amino acids at the C-terminus), together with a TP-rich linker (SRGGGTATPTPTPTPTP) at the C-terminus, was synthesized by NZYTech (Lisbon, Portugal) after codon optimization for *Pichia pastoris* expression. The synthesized CBM sequence was fused with that of Y300A in pPICZαA. The fusion also contains a His_6_-tag at the C-terminus to facilitate purification. DNA sequences were confirmed at the Center for Applied Genomics (the Hospital for Sick Children, Toronto). The synthetic gene was deposited in the GenBank database under the accession number of KC707634.

### His-tag purification of *Ct*CBM22A_Y300A

The confirmed plasmid was transformed into *P. pastoris* KM71H following the manufacturer’s instructions (Life Technologies, USA). Expression screening by immuno-colony blot, cell growth, protein expression and purification were conducted following previously reported methods^23^. Briefly, cells were grown at 15 °C and 250 rpm for 5 days, and 0.5% methanol was added every 24 h to induce recombinant protein expression. Culture supernatant was desalted and mixed with Ni-NTA resin (Qiagen, USA), bound proteins were then eluted with 250 mM imidazole, followed by centrifugal buffer exchange with 50 mM Tris-HCl pH 8.0.

Protein concentration was measured by the Bradford method (Bio-Rad Laboratories, USA), and verified by band densitometry on SDS-PAGE gels using known quantities of bovine serum albumin as a reference and ImageJ for image analysis (http://rsbweb.nih.gov/ij/).

### OSX purification of *Ct*CBM22A_Y300A

After five days of induction with 0.5% methanol, 250 mL *P. pastoris* culture supernatants comprising *Ct*CBM22A_Y300A were harvested by centrifugation for 10 min at 2,000 × g (repeated three times), then filtered through a 0.45 μm membrane. Half of the supernatant was then exchanged to 50 mM Tris-HCl pH 8.0 using 30 kDa MWCO Vivaspin 20 centrifugal concentrators (GE healthcare, UK). Insoluble OSX was then added at 1.25% (w/v) to 40 mL filtered supernatant and buffer exchanged supernatant. Resulting solutions were mixed for 24 h at 4 °C using a 360-degree rotator set at 8 rpm. Protein-bound OSX and unbound (flow-through) fractions were then collected by vacuum-filtration using a 0.45 μm membrane. Protein-bound OSX was washed 3 times with 50 mM Tris-HCl pH 8.0 and then stored in this buffer. All fractions were kept at 4 ^o^C.

### OSX binding efficiency

The protein in 700 μL of filtered supernatant, buffer-exchanged supernatant or flow-through fractions after OSX addition was separately precipitated with 10% trichloroacetic acid. The precipitate was washed thoroughly with ice-cold acetone, and the pellet was suspended in 40 μL of SDS loading dye. The proteins in 35–40 mg wet weight of bound OSX were extracted with 40 μL of the denaturing solution (10% SDS and 10% beta-mercaptoethanol) for 10 min at 100 ^o^C. Precipitated as well as extracted proteins were then analyzed using 10% SDS-PAGE gels.

The effect of Ca^2+^ on *Ct*CBM22A binding to xylan was investigated by incubating the fusion enzyme with OSX overnight at 4 ^o^C in the presence of 5 mM CaCl_2_; the amount of unbound and bound protein was then evaluated weekly by SDS-PAGE.

### Binding affinity on soluble polymeric substrates

Binding of *Ct*CBM22A_Y300A on soluble OSX, arabinoxylan, beech wood xylan, wheat bran hemicellulose and propoxylated wheat bran hemicellulose was evaluated by affinity gel electrophoresis^24^. Briefly, 0.01% of each polysaccharide was added to 7.5% (w/v) acrylamide in a Tris-glycine buffer (25 mM Tris, 250 mM glycine pH 8.3) prior to polymerization to produce native polyacrylamide gels. Protein samples (5 μg) were loaded into each gel followed by electrophoresis using ice-cold Tris-glycine buffer.

### Specific activity, enzyme kinetics and thermostability measurements

The production of oxidized sugars was determined by continuously measuring the co-production of H_2_O_2_ at 37 °C for 15 min using a chromogenic horseradish peroxidase assay^23^. Activity measurements were performed using 16 nM of each GOOX variant and 0.5 mM substrate; kinetic measurements were performed using the following substrate concentrations: 0.05–300 mM glucose; 0.05–1,200 mM xylose; 0.05–20 mM cellobiose; 0.01–10 mM cellotriose, xylobiose and xylotriose; 0.01–4 mM of longer cello- and xylo-oligosaccharides. GraphPad Prism5 software (GraphPad Software, USA) was used for curve fitting and deriving kinetic parameters.

To compare activities of GO and GOOX variants, 24 nM of each enzyme were separately assayed in triplicate with 10 mM of xylose, maltose and cellobiose; 1 mM of glucose and cellotetraose; and 0.6 g/L of mixed XOS. GOOX enzymes were assayed in 50 mM Tris-HCl pH 8.0 while GO was assayed in 50 mM sodium acetate pH 5.0.

Thermostability tests were performed by incubating GOOX enzymes (16 nM) at temperatures between 30 and 54 °C for 1 h, and then measuring residual activity (initial rate) on 0.5 mM cellobiose.

### H_2_O_2_ inactivation and stability

H_2_O_2_ inactivation of GOOX was investigated by incubating 0.1 μg of each GOOX variant (5–7 nM) at 37 °C in the dark in 250 μL solutions of 50 mM Tris-HCl (pH 8.0) containing 1 mM glucose or 0.5 mM cellobiose, and between 0–200 mM H_2_O_2_. The effect of H_2_O_2_ on GO activity was similarly determined, except that the buffer was replaced by 50 mM sodium acetate (pH 5.0) and 1 mM glucose was used. Substrate concentration was kept low to avoid any contribution from the de novo release of H_2_O_2_. Following 5-h incubation with glucose, or 2-h incubation with cellobiose, reactions were filtered through 10 K Omega^TM^ Nanosep centrifugal devices (Pall Corp., USA) to separate reaction products from each enzyme. To facilitate the high-performance anion-exchange chromatography (HPAEC) analyses, 100 U catalase was added to reactions containing glucose to prevent H_2_O_2_ interference, and then incubated for 1 h at 25 °C before filtration through 10 K Omega^TM^ Nanosep centrifugal devices.

The production of oxidized sugars was analyzed by HPAEC with pulsed amperometric detection (HPAEC-PAD) using a CarboPac PA1 (2 × 250 mm) analytical column (Dionex, USA) in an ICS5000 HPAEC-PAD system (Dionex, USA). The HPAEC-PAD samples were eluted at 0.25 mL/min using sodium acetate gradient (0–1,000 mM) in 100 mM NaOH^23^. Chromatograms were viewed and analyzed using Chromeleon 7.2 (Dionex, USA).

To test for stability, 16 nM GOOX enzymes and GO were also pre-incubated with 200 mM H_2_O_2_ for 30 min at 25 °C in the dark in an Eppendorf ThermoMixer with shaking (300 rpm). The H_2_O_2_ was then degraded using catalase (2 × 100 U) prior to addition of 1 mM glucose to measure residual activity. Following a 2-h incubation with glucose at 37 °C, each sample was filtered using a 10 K Omega^TM^ Nanosep centrifugal device prior to analysis by HPAEC-PAD.

Potential effects of residual cultivation media on H_2_O_2_ decomposition was tested in triplicate by incubating 1 μL of culture supernatant or medium with 74 μL of different H_2_O_2_ concentrations (up to 150 μM) in 96-well plates at 25 °C for 5 h in the dark. The remaining amount of H_2_O_2_ was measured using the chromogenic horseradish peroxidase assay.

### Total amino acid analysis

Both Y300A and *Ct*CBM22A_Y300A (1,000 pmoles) were incubated with 200 mM H_2_O_2_ in the presence of 1 mM glucose for 5 h in the dark. The samples were then washed intensively with water using a 10 K Omega^TM^ Nanosep centrifugal device, and treated with 6 N HCl containing 1% phenol for 24 h at 110 °C. The amino acid composition was analyzed at the SPARC BioCentre (the Hospital for Sick Children, Toronto).

## Results and Discussion

### Broader substrate specificity of Y300A

**Figure 1:**
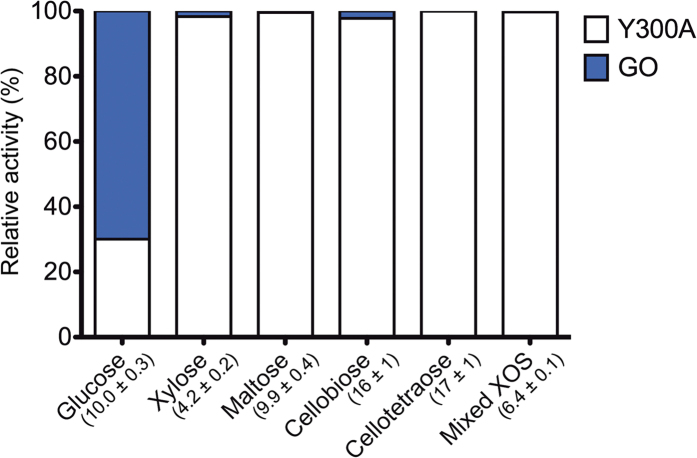
Relative activity of Y300A and GO on selected mono- and oligo-saccharides. Each of the following substrates was assayed in triplicate for both enzymes: 1 mM of glucose and cellotetraose; 10 mM of xylose, maltose and cellobiose; and 0.6 g/L of mixed xylo-oligosaccharides (XOS). The sum of Y300A and GO activity (U/mg) towards each substrate is indicated in parentheses, and shaded bars indicate the proportion of that activity attributed to Y300A (white) versus GO (blue).

The GOOX variant Y300A displays a similar substrate profile to the wild-type enzyme; however, it demonstrates higher activity on glucose^16^ and lower substrate inhibition^19^. Therefore, Y300A was chosen for the current comparative analysis with GO. The substrate preference of GO and Y300A was assayed using glucose, xylose, maltose, cellobiose, cellotetraose, and XOS. On tested mono- and oligosaccharides, GO exhibited only 2.5% activity relative to Y300A, with the exception of glucose, for which GO activity was 100% higher than Y300A (Fig. 1). The different substrate preference of Y300A and GO is likely attributed to the open active site of GOOX^25^ in comparison with the narrow binding pocket of GO, which is specifically suited for glucose^26^.

### Improving glucose oxidation by Y300A

**Figure 2:**
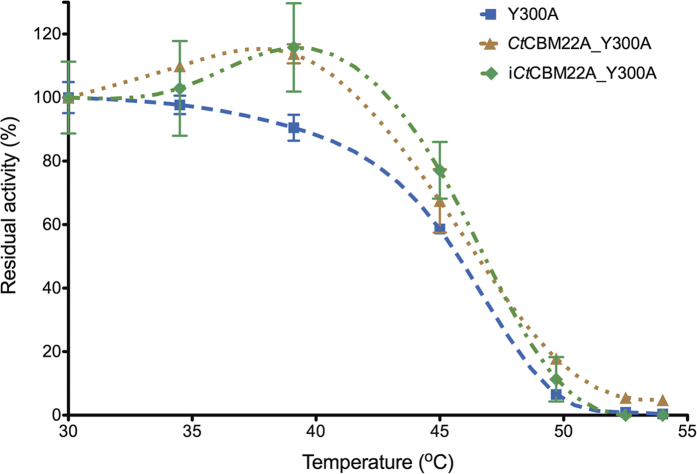
Thermostability of Y300A variants. Enzymes (16 nM) including OSX-immobilized *Ct*CBM22A_Y300A (or i*Ct*CBM22A_Y300A) were incubated at temperatures between 30 and 54 °C for 1 h, and residual activity was measured using 0.5 mM cellobiose.

The yield of *Ct*CBM22A_Y300A recombinantly expressed in *P. pastoris* KM71H was approximately 200 mg/L. Fusing *Ct*CBM22A to Y300A also increased *k*_cat_ values of the enzyme on all sugars, up to 67% in the case of cellotriose, from 670 min^−1^ to 1,120 min^−1^ (Table 1). A similar effect was previously seen when *Ct*CBM22A was fused to the N-terminus of the wild-type GOOX^23^. The *K*_m_ values of *Ct*CBM22A_Y300A on monosaccharides including glucose and xylose were also decreased by around 45%, resulting in higher catalytic efficiency (Table 1). For instance, *k*_cat_/*K*_m_ values of *Ct*CBM22A_Y300A on glucose and xylose (173 and 27 mM^−1^ min^−1^, respectively) are about 100% higher than those of Y300A (Table 1), and more than 600% higher than those reported for wild-type GOOX^19^. As *Ct*CBM22A does not bind xylose and glucose^27^, the observed improvement in *K*_m_ and catalysis on monosaccharides, as well as slight thermal activation at 40 °C (Fig. 2) by *Ct*CBM22A_Y300A, is likely due to a subtle structural change induced in the Y300A variant upon fusion to *Ct*CBM22A, as previously proposed for GOOX^23^.

As expected, the catalytic efficiency of the fusion enzyme on glucose was lower than those reported for *A. niger* GO (i.e., 1,800^28^ and 2,280 mM^−1^ min^−1^ 29). However, the significantly lower *K*_m_ values of *Ct*CBM22A_Y300A on glucose (5.4 mM) compared to those reported for GO (~ 30^28^ and ~50 mM^29^) may offer advantages to applications containing low glucose concentrations.

### Enhanced xylan binding through *Ct*CBM22A fusion

**Figure 3:**
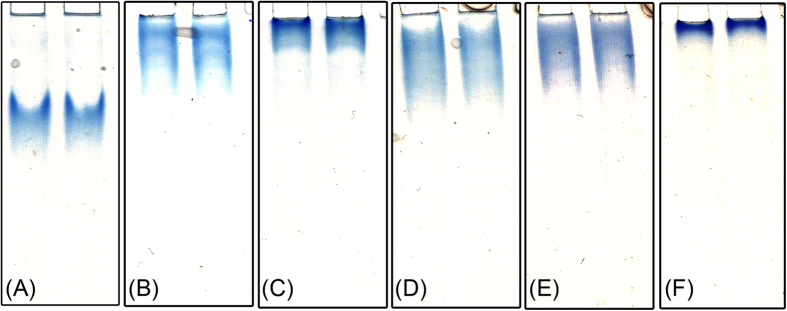
Binding of the *Ct*CBM22A_Y300A fusion on polymeric substrates. (**A**) Control, no polysaccharide; (**B**) Arabinoxylan; (**C**) Soluble oat spelt xylan; (**D**) Beech wood xylan; (**E**) Wheat bran hemicellulose; (**F**) Propoxylated wheat bran hemicellulose. Polysaccharides (0.01%) were added to 7.5% native polyacrylamide gels, and 5 μg of *Ct*CBM22A_Y300A were loaded in duplicate; the figure was composed from six separate gels.

The presence of different xylans including OSX, arabinoxylan, beech wood xylan and wheat bran xylan significantly reduced the migration of *Ct*CBM22A_Y300A in native polyacrylamide gels, indicating strong xylan binding by the fusion enzyme (Fig. 3). Reduced migration was more obvious on buffer-soluble oat spelt xylan and propoxylated wheat bran xylan, as shown by sharp bands at or near the loading wells (Fig. 3). There was no difference in migration between the *Ct*CBM22A fusion of Y300A (Fig. 3) and the previously reported wild-type fusion^23^, suggesting that the tyrosine substitution did not impact hemicellulose binding.

### Simultaneous purification and immobilization using dietary fibre

Enzyme purification at large scale is expensive and labor-intensive. Given that *Ct*CBM22A fusion was previously shown to immobilize wild-type GOOX on xylan^23^, insoluble OSX was chosen for ability to immobilize and recover *Ct*CBM22A_Y300A directly from culture supernatant while providing additional dietary fibre in food applications^30^.

**Figure 4:**
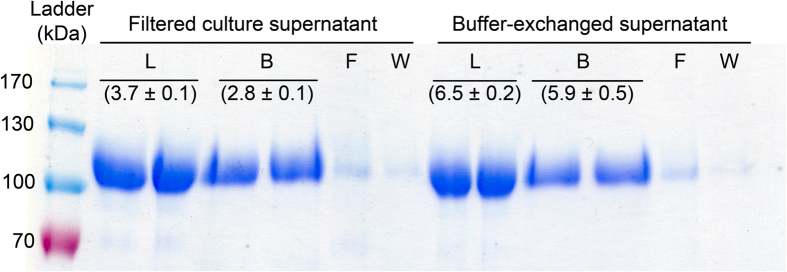
Binding and specific activity of different *Ct*CBM22A_Y300A preparations. The *Ct*CBM22A_Y300A (L fraction) from the filtered culture supernatant or the supernatant after buffer-exchange was mixed with insoluble OSX and then filtered; the amount of protein that was in the flow-through (F fraction) or in subsequent washing steps (W fraction) as well as that bound to OSX (B fraction) was analyzed. Specific activity (U/mg) of L and B fractions on cellobiose is indicated in parentheses.

Insoluble OSX was directly added to filtered culture supernatant containing *Ct*CBM22A_Y300A or the supernatant that had been exchanged to 50 mM Tris buffer pH 8.0. The amount of protein that remained bound to insoluble OSX (B fraction) was then evaluated by SDS-PAGE (Fig. 4). The original amount of protein in supernatant solutions before loading (L fraction), those in the first flow-through (F fraction) and flow-through during washing (W fraction) were also analyzed. Low amounts of protein in flow-through solutions were detected, indicating that most of the protein bound to OSX (Fig. 4). Furthermore, there was a similar amount of bound protein from filtered culture supernatant and buffer-exchanged supernatant samples, suggesting that the culture supernatant did not interfere with OSX binding of *Ct*CBM22A_Y300A. Therefore, OSX can be used to recover *Ct*CBM22A_Y300A directly from culture supernatant without buffer-exchange, facilitating the simultaneous purification and immobilization of the enzyme.

Although the filtered, spent culture did not impact *Ct*CBM22A_Y300A binding to OSX, enzyme activity towards cellobiose was reduced by approximately 50% (Fig. 4). Fresh induction media did not interfere with the assay (Fig. S1), which suggests that the reduced activity resulted in part by components released by the Pichia transformant.

Further analysis of *Ct*CBM22A_Y300A from buffer exchanged culture medium and immobilized to OSX confirmed that immobilization did not reduce enzyme activity on cellobiose (Fig. 4). This suggests that cellobiose did not compete with OSX for *Ct*CBM22A binding, which is consistent with the high *K*_a_ of *Ct*CBM22A towards xylo-oligosaccharides and particularly OSX^27^, and with our earlier characterization of *Ct*CBM22A_GOOX^23^.

Binding between *Ct*CBM22A_Y300A and OSX remained stable in 2 M of NaCl, even after 12 weeks at 4 °C (data not shown). However, up to 10% of the protein desorbed from OSX during storage at room temperature, of which about 50% bound back to the OSX upon the addition of 5 mM CaCl_2_ (Fig. S2). Calcium probably stabilizes the functional form of *Ct*CBM22A, as predicted from structural characterizations of *Ct*CBM22A^27^.

### H_2_O_2_ tolerance of GOOX variants and GO

The impact of H_2_O_2_ on GO activity and on activities of GOOX variants Y300A, *Ct*CBM22A_Y300A and OSX-immobilized *Ct*CBM22A_Y300A (i*Ct*CBM22A_Y300A), was compared using glucose as the substrate.

**Figure 5:**
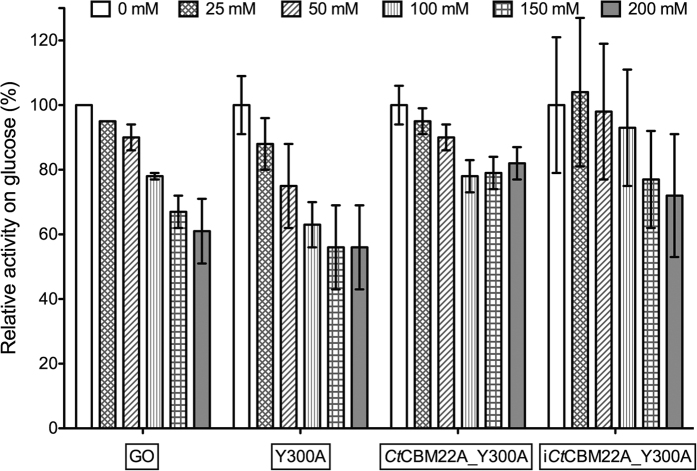
H_2_O_2_ tolerance of GOOX variants and GO. Enzymes (0.1 μg) were incubated with 1 mM glucose and different concentrations of H_2_O_2_ (up to 200 mM). The production of gluconic acid was then quantified by HPAEC-PAD.

Fusion of *Ct*CBM22A to Y300A appeared to increase Y300A stability in H_2_O_2_, as *Ct*CBM22A_Y300A and i*Ct*CBM22A_Y300A retained approximately 80% activity after 5 h in 200 mM H_2_O_2_ (Fig. 5). The higher turnover number and better glucose binding by the fusion enzyme (Table 1) might have outweighed activity losses resulting from H_2_O_2_ inactivation. No significant difference in H_2_O_2_ tolerance was observed between Y300A and GO (Fig. 5), which displayed decreasing glucose oxidation with increasing H_2_O_2_ concentration and retained approximately 60% activity at 200 mM H_2_O_2_. It should be noted, however, that the commercial GO preparation used herein contains a very low but detectable amount of catalase (≤0.1 U/mg of GO, as reported by Sigma). An earlier report shows that GO retains approximately 20% activity after 20-min incubation with 100 mM H_2_O_2_^12^. This difference in H_2_O_2_ tolerance of GO samples may reflect differences in activity measurement (i.e., oxygen consumption versus gluconic acid production herein) or improvement in commercial preparations of GO.

**Figure 6:**
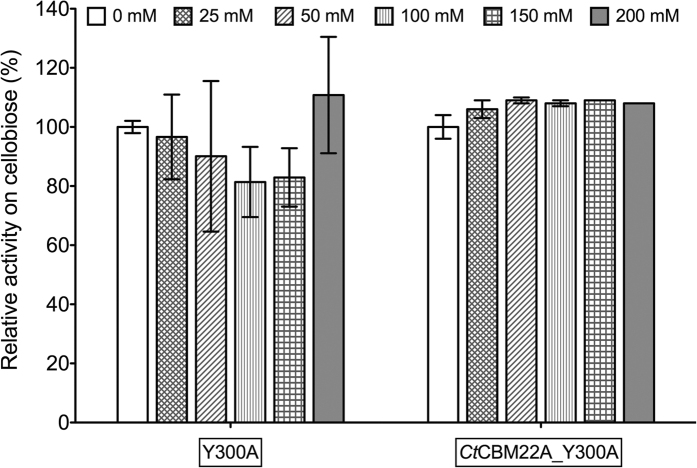
Cellobiose oxidation by Y300A and *Ct*CBM22A_Y300A in the presence of different amounts of H_2_O_2_. Relative activity of 16 nM GOOX variants on 0.5 mM cellobiose in the presence of H_2_O_2_ (0–200 mM). Activity values were derived from HPAEC-PAD quantification of reaction products.

When retaining the same reaction conditions but substituting glucose for cellobiose, H_2_O_2_ inactivation was not detected for any GOOX variant (Fig. 6). The catalytic efficiency of Y300A and *Ct*CBM22A_Y300A on cellobiose (3,400 and 4,000 min^−1^ mM^−1^, respectively) is an order of magnitude higher than that on glucose (98 and 173 min^−1^ mM^−1^, respectively) (Table 1). Presumably then, GOOX variants were able to oxidize its preferable substrate, cellobiose, before H_2_O_2_ inactivation took effect.

**Figure 7:**
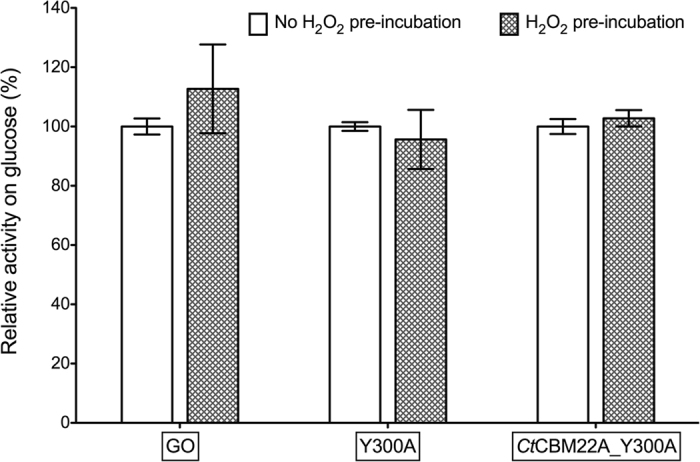
Residual activity of GO, Y300A and *Ct*CBM22A_Y300A on glucose following pre-incubation with H_2_O_2_. After a 30-min pre-incubation with 200 mM H_2_O_2_ at 25 °C, H_2_O_2_ was removed and 1 mM glucose was added; the reaction was then incubated for 2 h at 37 °C before HPAEC-PAD analysis for the production of gluconic acid.

Pre-incubation of GO and GOOX variants with 200 mM H_2_O_2_ for 30 min prior to addition of glucose did not impact enzyme activity (Fig. 7). This suggests that reduced forms of GO and GOOX are more susceptible to inactivation by H_2_O_2_, as previously reported for GO^12^.

### Apparent mode of H_2_O_2_ inactivation

The oxidation of key methionine residues in the presence of H_2_O_2_ has been proposed to cause GO inactivation^12^. The total amino acid analysis of GOOX variants, however, revealed significant loss of histidine in Y300A and its *Ct*CBM22A fusion following incubation with H_2_O_2_ (Table S1). The broader active site as well as the double-covalent flavinylation in GOOX might contribute to this potential difference in H_2_O_2_ inactivation. Notably, histidine residues H70 and H138 are positioned in the GOOX active site, of which the N^δ1^ atom of H70 binds to the 8α-methyl group of the isoalloxazine ring of FAD while H138 interacts with FAD via a hydrogen bond (Fig. S3). The oxidation of either these histidine residues would affect FAD binding and electrochemical properties. For instance, an H70A substitution, which led to removal of the 8α -N1-histidyl bond, retained only 0.5% catalytic efficiency and about 55% redox potential, compared with the wild-type^31^. Histidine residues can be oxidized by H_2_O_2_ to form a mixture of products including Asp and 2-oxo-histidine^32^. Since H_2_O_2_ treatment only slightly increased the (Asp + Asn) content in Y300A and *Ct*CBM22A_Y300A samples (Table S1), 2-oxo-histidine is probably the major product of oxidation. As histidine loss was 6.7 and 9.3 mol/mol of protein in Y300A and its *Ct*CBM22A fusion, respectively (Table S1), other histidine residues were also modified. Besides the two exposed histidine residues in the active site and six in the 6x-his tag, Y300A also has four histidine residues on the surface, and the fusion provides two additional exposed histidines. Loss of tyrosine in the H_2_O_2_-treated samples was also detected, particularly in *Ct*CBM22A_Y300A, which has three tyrosine residues in the binding cleft out of its eleven surface-exposed tyrosine residues.

## Conclusion

GOOX variants Y300A and *Ct*CBM22A_Y300A demonstrate potential benefit in applications characterized by relatively high H_2_O_2_ concentrations and mixed sugars. Both Y300A and *Ct*CBM22A_Y300A have broader substrate profiles than the commonly used GO, and *Ct*CBM22A_Y300A displayed less H_2_O_2_ inactivation than GO when assayed with glucose. Furthermore, both GOOX variants showed no H_2_O_2_ inactivation on cellobiose, their favorable substrate. *Ct*CBM22A_Y300A is the best variant of GOOX to date for glucose oxidation (as well as xylose oxidation) while retaining considerable activity on oligosaccharides. The presence of *Ct*CBM22A also permits enzyme binding to OSX, facilitating simultaneous purification and immobilization of the enzyme, and ancillary fibre enrichment in potential food applications.

## Additional Information

**How to cite this article**: Vuong, T. V. *et al*. Direct comparison of gluco-oligosaccharide oxidase variants and glucose oxidase: substrate range and H_2_O_2_ stability. *Sci. Rep.* **6**, 37356; doi: 10.1038/srep37356 (2016).

**Publisher’s note:** Springer Nature remains neutral with regard to jurisdictional claims in published maps and institutional affiliations.

## Supplementary Material

Supplementary Information

## Figures and Tables

**Table 1:** Kinetic parameters of *Ct*CBM22A_Y300A on mono- and oligo-saccharides.

	Y300A^a^			*Ct*CBM22A*_*Y300A		
	*k*_cat_ (min^−1^)	*K*_m_ (mM)	*k*_cat_/*K*_m_	*k*_cat_ (min^−1^)	*K*_m_ (mM)	*k*_cat_/*K*_m_
Glucose	793 ± 14	8.1 ± 0.4	98 ± 5	941 ± 8	5.4 ± 0.2	173 ± 7
Cellobiose	823 ± 16	0.25 ± 0.02	3,400 ± 300	980 ± 20	0.25 ± 0.02	4,000 ± 400
Cellotriose	670 ± 10	0.25 ± 0.02	2,700 ± 200	1,120 ± 20	0.21 ± 0.02	5,300 ± 500
Cellopentaose	nd	nd	nd	1,100 ± 20	0.30 ± 0.02	3,600 ± 300
Cellohexaose	nd	nd	nd	990 ± 20	0.31 ± 0.02	3,200 ± 300
Xylose	680 ± 8	51.8 ± 1.9	13.1 ± 0.5	975 ± 19	37 ± 3	27 ± 2
Xylobiose	797 ± 7	5.11 ± 0.15	156 ± 5	1,183 ± 16	5.84 ± 0.15	202 ± 6
Xylotriose	832 ± 7	3.15 ± 0.11	260 ± 10	1,180 ± 20	4.57 ± 0.16	259 ± 10
Xylopentaose	nd	nd	nd	1,570 ± 30	4.49 ± 0.15	349 ± 14
Xylohexaose	nd	nd	nd	1,046 ± 19	3.0 ± 0.1	353 ± 13

^a^From Foumani *et al*.^16^; ^nd^Not determined.
