# Supplementary material for: Direct comparison of gluco-oligosaccharide oxidase variants and glucose oxidase: substrate range and H2O2 stability
Source: Sci Rep. 2016 Nov 21;6:37356. doi: 10.1038/srep37356 (PMC5116756; doi:10.1038/srep37356)

Direct comparison of gluco-oligosaccharide oxidase variants and glucose oxidase: substrate range and H_2O_2 stability

Thu V. Vuong, Maryam Foumani, Benjamin MacCormick, Rachel Kwan and Emma R. Master

Supplemental tables

Table S1: Amino acid analysis of Y300A and its fusion *Ct*CBM22A_Y300A

		Calculated composition of Y300A[a]				Calculated composition of fusion		
	Y300A composition	Untreated Y300A	Treated Y300A	Treated Y300A - Untreated Y300A	Fusion composition	Untreated fusion	Treated fusion	Treated fusion - Untreated fusion
Asp+Asn[b]	65	65.4	66.5	1.2	83	83.2	84.9	1.7
Glu+Gln	52	55.6	54.4	-1.2	68	71.8	70.8	-1.0
Ser	26	27.0	26.4	-0.6	37	38.6	38.3	-0.3
Gly	47	49.2	49.7	0.5	65	70.5	71.0	0.5
His	19	18.8	12.2	-6.7	21	20.5	11.3	-9.3
Arg	16	16.2	15.8	-0.4	25	23.0	20.9	-2.1
Thr	29	24.4	26.7	2.3	50	45.8	46.4	0.7
Ala	49	49.7	48.6	-1.2	64	63.8	66.2	2.4
Pro	13	13.4	14.0	0.6	26	28.2	29.3	1.1
Tyr	25	24.8	23.3	-1.6	38	34.1	29.6	-4.5
Val	29	28.4	26.9	-1.0	42	40.2	41.4	1.2
Met	7	6.4	6.8	0.4	10	10.2	8.8	-1.4
Ile	23	20.6	19.6	-1.0	30	26.7	27.6	0.9
Leu	41	38.8	38.8	0.1	48	45.4	46.2	0.8
Phe	21	21.8	22.7	0.9	28	28.5	28.9	0.3
Lys	21	19.0	19.8	0.8	25	24.8	23.5	-1.2

[a] Treated proteins were incubated with 200 mM H_2O_2 for 5 h in the presence of 1 mM glucose.

[b] Asp and Asn were merged into one peak, Glu and Gln were merged as well; where as Cys and Trp peaks could not be detected.

Supplemental figure legend

Figure S1: Effect of Pichia culture supernatant on the H_2O_2-based colorimetric assay. The colorimetric assays were conducted at 25 ^{o}C with different concentrations of H_2O_2 (up to 150 μM) alone or in the presence of either fresh medium, negative control culture supernatant (i.e. Pichia with a negative control vector), *Ct*CBM22A_Y300A culture supernatant or its filtrate after a 10 K cut-off membrane filtration.

Figure S2: Calcium effect on binding recovery. Around 50 % of leaked *Ct*CBM22A_Y300A from filtered culture supernatant (CS) and the supernatant after buffer-exchange (BX) bound back to OSX in the addition of 5 mM $CaCl_2$, as calculated by band densitometry analysis of SDS-PAGE gels.

Figure S3: The active site of a GOOX variant. The cofactor FAD has a covalent bond with H70 and a hydrogen bond (shown as red dashes) with H138; the substrate analog ABL in the active site (PDB ID: 2AXR) was also shown.

Figure S1

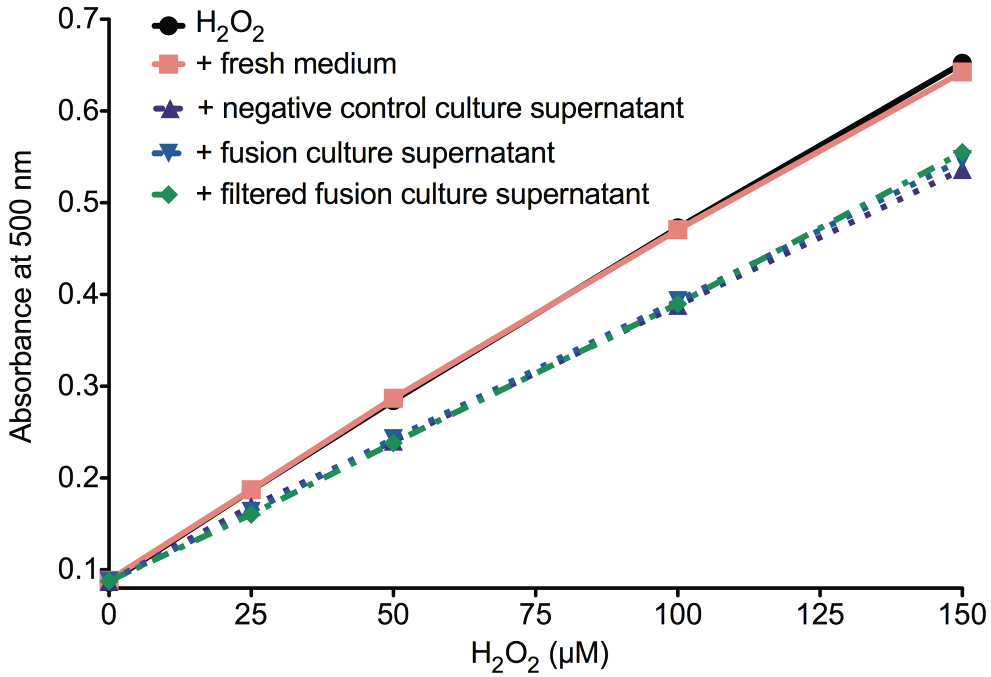

0.7
0.6
0.5
0.4
0.3
0.2
0.1
Absorbance at 500 nm
0
25
50
75
100
125
150
H2O2 (μM)
H2O2
+ fresh medium
+ negative control culture supernatant
+ fusion culture supernatant
+ filtered fusion culture supernatant

Figure S2

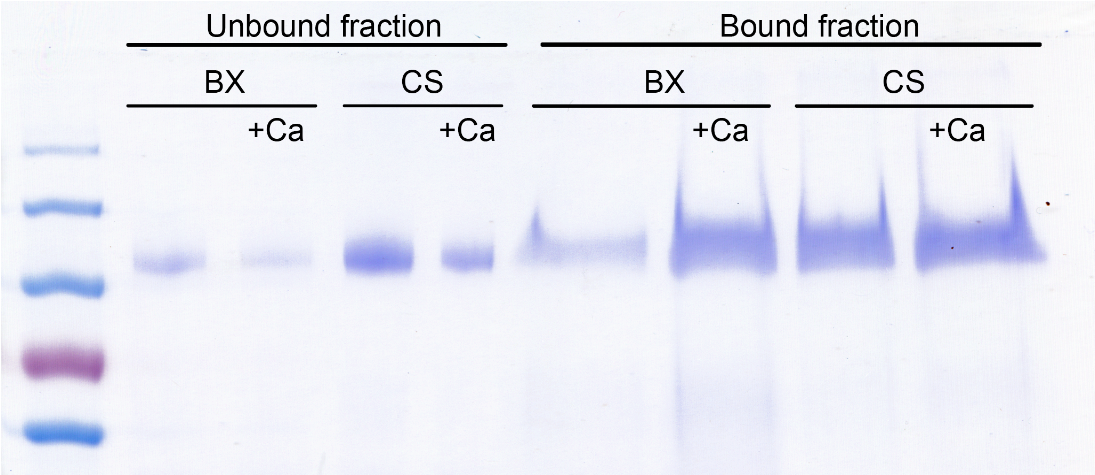

Figure S3

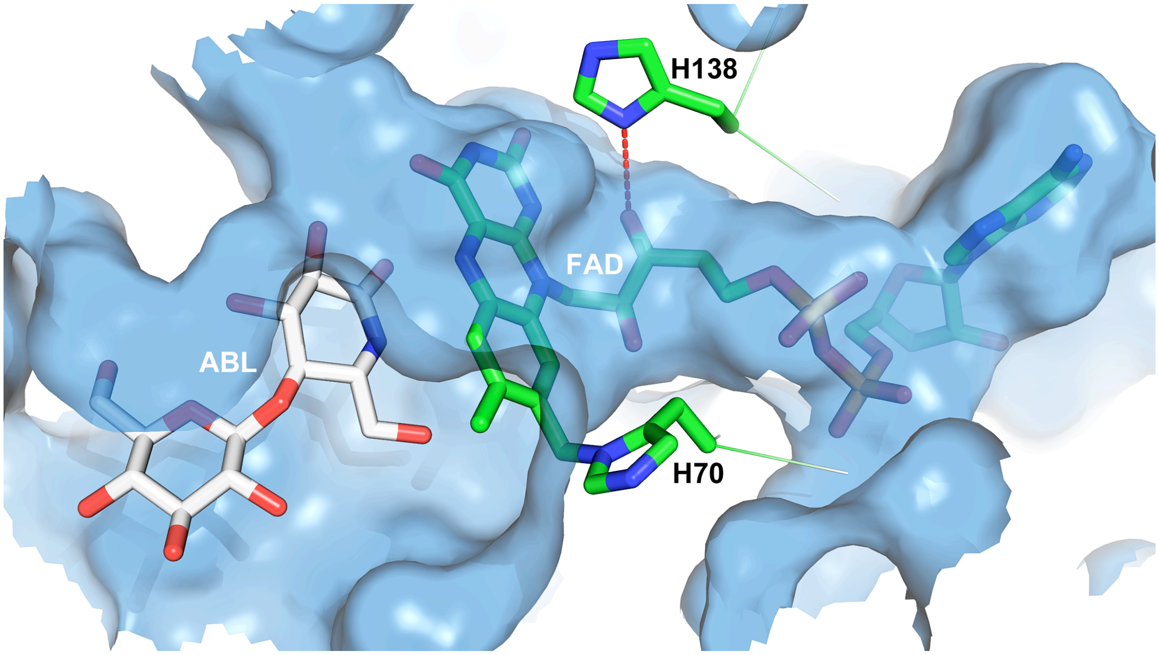

Supplement: Supplementary Information [file srep37356-s1.pdf]
